# From pose estimation accuracy to motor biomarker reliability: a reliability-centred evaluation framework for markerless movement analysis

**DOI:** 10.3389/fninf.2026.1790241

**Published:** 2026-10-01

**Authors:** Valentin Fernandez, Khabiba Abdiyeva, Sébastien Brot, Luis Perreira de Almeida, Afsaneh Gaillard, Christine Fernandez-Maloigne

**Affiliations:** 1 LNEC, INSERM U1084, Université de Poitiers, Poitiers, France; 2 CNC, Center for Neuroscience and Cell Biology, University of Coimbra, Coimbra, Portugal; 3 Department of Software Engineering, Samarkand State University, Samarkand, Uzbekistan; 4 XLIM, UMR CNRS 7252, Université de Poitiers, Poitiers, France

**Keywords:** markerless pose estimation, motor biomarkers, kinematic analysis, reliability, robustness, neuroinformatics

## Abstract

Markerless pose estimation has become an essential tool for quantitative movement analysis in neuroscience. However, current evaluation frameworks primarily rely on frame-wise spatial accuracy metrics, which provide only limited information about the reliability of the downstream kinematic biomarkers derived from reconstructed trajectories. In this study, we propose a reliability-centred evaluation framework designed to assess the preservation of downstream kinematic information in pose estimation pipelines. The framework was evaluated exclusively using DeepLabCut-based architectures trained and tested under identical experimental conditions. Forelimb trajectories of mice performing the Montoya staircase task were analysed using nine DeepLabCut-based convolutional neural network architectures. We introduce the Motor Biomarker Reliability Composite Score (MBRCS), which integrates four complementary dimensions of trajectory reliability: spatial accuracy, temporal stability, occlusion robustness, and kinematic fidelity. Each component was normalized to a 0–1 scale, where higher values indicate greater trajectory reliability. Although several architectures achieved comparable frame-wise localisation performance, larger differences were observed in temporal stability, occlusion robustness and downstream kinematic fidelity. Across the evaluated architectures, MBRCS values spanned almost the entire normalized range (0.00–0.99). These differences were reflected in the Normalized Jerk Score (NJS), which was used as the downstream kinematic biomarker in this study. Overall, our findings suggest that frame-wise localisation accuracy alone may not fully capture differences in trajectory reliability that become apparent when temporal stability, occlusion robustness and kinematic fidelity are jointly considered. The proposed framework provides a structured and reproducible methodology for benchmarking pose estimation pipelines according to the reliability of downstream motor biomarkers.

## 1 Introduction

Quantitative movement analysis is a fundamental component of both preclinical and clinical neuroscience, enabling the objective assessment of motor impairment, recovery, and therapeutic efficacy. Beyond conventional endpoint-based behavioural evaluations, kinematic analysis provides a more detailed characterization of movement quality through descriptors such as coordination, variability, and smoothness, which reflect underlying neural control mechanisms and compensatory strategies (Whishaw et al., 1992; Flash and Hogan, 1985; Hogan and Sternad, 2009). These quantitative biomarkers are particularly valuable when apparent behavioural recovery coexists with persistent alterations in movement organisation.

Recent advances in deep learning have considerably expanded the capabilities of quantitative movement analysis through markerless pose estimation, enabling the non-invasive extraction of anatomical trajectories from video recordings (Mathis et al., 2018; Pereira et al., 2019; Lauer et al., 2022; Graving et al., 2019). Frameworks such as DeepLabCut have demonstrated high-precision tracking of user-defined anatomical landmarks and are now widely used in neuroscience and behavioural research (Mathis et al., 2018). Consequently, markerless pose estimation has become an important component of modern neuroinformatics and movement analysis pipelines.

Despite these advances, the evaluation of pose estimation pipelines still relies predominantly on frame-wise spatial localisation metrics, including root mean square error (RMSE), percentage of correct keypoints (PCK), and mean average precision (mAP) (Mathis et al., 2018; Pereira et al., 2019; Lauer et al., 2022). Although these metrics provide valuable information on localisation performance, they offer only a partial assessment of the reliability of downstream kinematic biomarkers derived from reconstructed trajectories. This limitation is particularly important for derivative-based quantities such as velocity and jerk, where temporal differentiation amplifies high-frequency tracking noise and frame-to-frame fluctuations. Consequently, pipelines exhibiting similar localisation accuracy may nevertheless differ in their ability to preserve reliable kinematic information.

From a measurement-theoretical perspective, reliability and validity represent complementary but distinct concepts (Koo and Li, 2016; Atkinson and Nevill, 1998; Portney and Watkins, 2009). In markerless pose estimation, reliability refers to the stability and consistency of reconstructed trajectories under perturbations such as temporal instability or occlusion, whereas validity concerns the extent to which the derived biomarkers preserve biologically meaningful differences between experimental conditions. Existing benchmarking studies rarely evaluate these complementary dimensions jointly, resulting in an incomplete characterisation of pose estimation performance for downstream movement analysis applications.

The aim of this study was therefore to develop a reliability-centred evaluation framework for markerless pose estimation pipelines. We propose a unified benchmarking framework integrating four complementary dimensions of trajectory reliability: spatial accuracy, temporal stability, occlusion robustness, and kinematic fidelity. The framework was evaluated using multiple DeepLabCut-based architectures applied to forelimb movements of mice performing the Montoya staircase task under identical experimental conditions. Finally, downstream functional discriminability analyses were conducted to determine whether trajectories with higher reliability better preserved biologically meaningful group differences.

## 2 Related work

### 2.1 Markerless pose estimation in biomedical movement analysis

Deep learning-based markerless pose estimation has become one of the principal approaches for quantitative movement analysis in neuroscience and behavioural research (Mathis et al., 2018; Pereira et al., 2019; Lauer et al., 2022; Graving et al., 2019; Marks et al., 2022; Kane et al., 2020; Mathis and Mathis, 2020). Frameworks such as DeepLabCut, SLEAP and DeepPoseKit automatically estimate anatomical landmarks from video recordings without the need for physical markers (Mathis et al., 2018; Pereira et al., 2019; Lauer et al., 2022; Graving et al., 2019). These methods have considerably expanded the possibilities for analysing movement in both preclinical and clinical settings by enabling the extraction of detailed kinematic trajectories from standard video data.

The reconstructed trajectories support the computation of numerous kinematic biomarkers, including movement smoothness, coordination, variability and inter-segment dynamics, which are sensitive indicators of motor function and neurological impairment (Whishaw et al., 1992; Flash and Hogan, 1985; Hogan and Sternad, 2009; Balasubramanian et al., 2015). As a result, markerless pose estimation has become an essential component of many contemporary movement analysis pipelines. Despite these advances, evaluation methodologies have evolved more slowly than pose estimation algorithms themselves.

### 2.2 Existing evaluation metrics for pose estimation

Current benchmarking strategies primarily evaluate pose estimation pipelines using frame-wise localisation metrics such as root mean square error (RMSE), percentage of correct keypoints (PCK), and mean average precision (mAP) (Mathis et al., 2018; Pereira et al., 2019; Lauer et al., 2022). These metrics provide standardized and reproducible measures of localisation accuracy and are widely adopted for comparing different pose estimation architectures.

However, localisation accuracy represents only one aspect of trajectory quality. Downstream movement analysis relies on continuous trajectories from which kinematic biomarkers are derived, making temporal consistency and trajectory integrity equally important for many biomedical applications.

### 2.3 Beyond spatial accuracy: toward reliable kinematic biomarkers

Several recent studies have highlighted the limitations of evaluating pose estimation exclusively through localisation accuracy (Balasubramanian et al., 2015; Seethapathi et al., 2019; Kidziński et al., 2020). Tracking noise, temporal jitter, uncertainty estimation and occlusion handling can substantially affect the quality of reconstructed trajectories and propagate to downstream kinematic analyses. This issue is particularly relevant for higher-order kinematic descriptors, such as velocity, acceleration and jerk, which are inherently sensitive to temporal perturbations.

Recent approaches have therefore investigated individual aspects of trajectory reliability, including temporal smoothness, uncertainty estimation, biomechanical plausibility and robustness to occlusions (Balasubramanian et al., 2015; Seethapathi et al., 2019; Kidziński et al., 2020). Although these studies represent important advances, they generally focus on individual properties rather than providing a unified framework for evaluating downstream biomarker reliability.

### 2.4 Remaining challenges and contribution of the present study

To our knowledge, no previous benchmarking framework jointly integrates spatial accuracy, temporal stability, occlusion robustness and kinematic fidelity into a single reliability-oriented evaluation framework for markerless pose estimation. Furthermore, few studies explicitly distinguish intrinsic trajectory reliability from downstream biological validation.

To address these limitations, we propose a reliability-centred evaluation framework that combines four complementary dimensions of trajectory reliability into the Motor Biomarker Reliability Composite Score (MBRCS). The framework is evaluated using multiple DeepLabCut-based architectures under identical experimental conditions and is complemented by downstream functional discriminability analyses to assess whether improvements in trajectory reliability translate into better preservation of biologically meaningful motor differences.

## 3 Reliability-centred benchmarking framework

### 3.1 Conceptual framework

Markerless pose estimation should be regarded as an intermediate stage within a complete movement analysis pipeline rather than as an end in itself. The ultimate objective is not merely the accurate localisation of anatomical landmarks, but the extraction of reliable kinematic biomarkers from reconstructed trajectories.

Tracking errors arising from spatial inaccuracies, temporal instability or occlusions may propagate through subsequent kinematic computations and alter the derived movement features. This issue is particularly relevant for derivative-based quantities such as velocity and jerk, for which temporal differentiation amplifies high-frequency tracking noise and frame-to-frame fluctuations (Flash and Hogan, 1985; Hogan and Sternad, 2009; Balasubramanian et al., 2015). Consequently, frame-wise localisation accuracy alone may not fully characterise the reliability of downstream kinematic biomarkers.

Within the proposed framework, trajectory reliability is described through four complementary dimensions: spatial accuracy (A), temporal stability (T), occlusion robustness (O), and kinematic fidelity (K). These dimensions characterise the intrinsic reliability of reconstructed trajectories. In contrast, downstream functional discriminability is considered an external validation step that evaluates whether reliable trajectories preserve biologically meaningful differences between experimental groups.

These four dimensions are integrated into a unified Motor Biomarker Reliability Composite Score (MBRCS), while downstream discriminability analyses are performed independently to assess the biological relevance of the reconstructed trajectories as illustrated on Figure 1.

**Figure 1:**
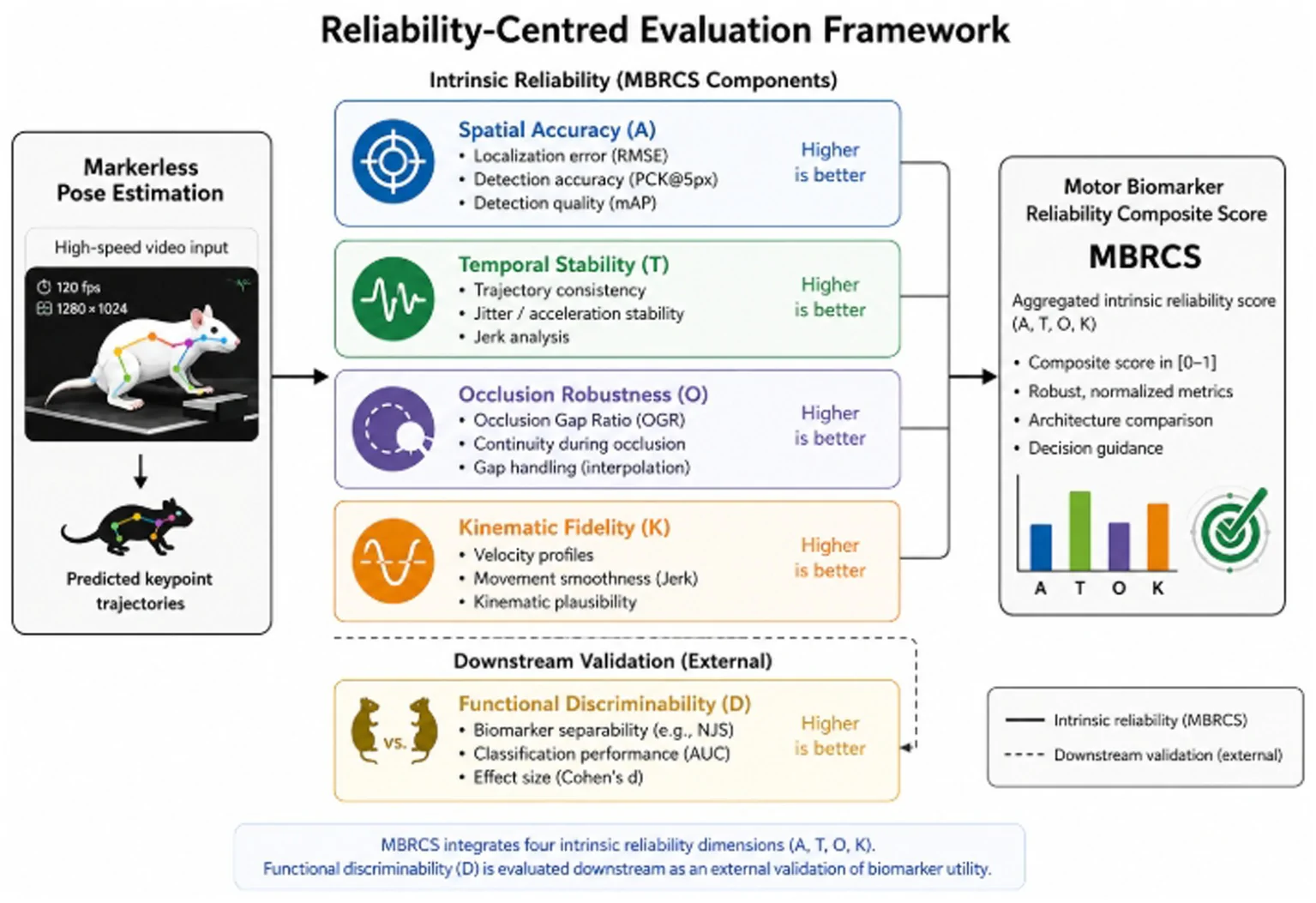
Reliability-centred Evaluation Framework for markerless pose estimation pipelines. This figure provides a conceptual overview of the proposed reliability-centred evaluation framework for markerless pose estimation in movement analysis. The framework distinguishes four intrinsic reliability dimensions, which are integrated into the Motor Biomarker Reliability Composite Score (MBRCS): spatial accuracy (A), temporal stability (T), occlusion robustness (O) and kinematic fidelity (K). Functional discriminability (D) is evaluated separately as an external validation component assessing the preservation of group-level motor differences. Predicted keypoint trajectories derived from markerless pose estimation are processed to extract kinematic biomarkers, which are then evaluated across different reliability dimensions to enable the comparative benchmarking of pose estimation pipelines.

### 3.2 Mathematical definition of the MBRCS

For each architecture *i*, let *m_i,j_* denote the raw metric of the evaluation dimension *j*. To make the different evaluation metrics comparable, we normalised all the values on a [0, 1] scale using robust, percentile-based normalisation:

$${\tilde{m}}_{i,j}=\text{clip}(\frac{m_{i,j}-q_{0.05,j}}{q_{0.95,j}-q_{0.05,j}},0,1)$$

where q_0.05,j_ and q_0.95,j_ denote the 5th and 95th percentiles of metric (j), respectively.

Percentile-based normalisation was selected to reduce sensitivity to extreme values and distributional heterogeneity across metrics with different numerical scales and statistical properties. Compared with conventional min-max normalization, percentile scaling provides greater robustness to outliers while preserving relative ranking consistency across architectures. Values falling outside the 5th-95th percentile interval were clipped to the [0,1] range. Consequently, normalized scores approaching 0 should not be interpreted as absence of performance, but rather as reflecting relative positioning near the lower bound of the evaluated distribution.

For cost-type metrics, where lower values suggest better performance (e.g., RMSE or trajectory instability measures), the normalized scores were inverted:

$${\tilde{m}}_{i,j}^{\mathrm{inv}}=1-{\tilde{m}}_{i,j}$$

The Motor Biomarker Reliability Composite Score (MBRCS) is defined as a weighted combination consisting of four complementary dimensions: spatial accuracy (A_i_), temporal stability (T_i_), occlusion robustness (Oi) and kinematic fidelity (Ki):

$${\text{MBRCS}}_{i}=w_{A}A_{i}+w_{T}T_{i}+w_{O}O_{i}+w_{K}K_{i}$$

with

$$\sum_{j}w_{j}=1,w_{j}\geq 0$$

A default weighting scheme is proposed:

$$\left(w_{A},w_{T},w_{O},w_{K},)=(0.15,0.30,0.25,0.30\right)$$

These weights were not optimized to maximize downstream performance but were defined *a priori* according to the expected contribution of each reliability dimension to downstream kinematic analyses. Greater weights were assigned to temporal stability and kinematic fidelity because derivative-based kinematic descriptors are particularly sensitive to temporal fluctuations and noise amplification.

To evaluate the robustness of the proposed weighting strategy, additional analyses were performed using alternative weighting configurations, including uniform weighting and accuracy-prioritised weighting. These analyses produced highly consistent architecture rankings (Spearman’s *ρ* > 0.9), indicating that the MBRCS is not strongly dependent on the precise weighting scheme.

The MBRCS is intended as a comparative benchmarking metric to facilitate the evaluation and selection of pose estimation pipelines for movement analysis applications rather than as an absolute measure of performance. Computational efficiency was therefore assessed separately and was intentionally not incorporated into the composite score.

### 3.3 Operational definition of components

#### 3.3.1 Spatial accuracy ( $A_{i}$ )

Spatial accuracy quantifies frame-wise localisation performance using three standard benchmarking metrics widely employed in pose estimation studies: root mean square error (RMSE), percentage of correct keypoints (PCK@5 px), and mean average precision (mAP) (Mathis et al., 2018; Pereira et al., 2019; Lauer et al., 2022). RMSE is, on average, the Euclidean distance between predicted and ground-truth keypoints:

$$\text{RMSE}=\sqrt{\frac{1}{N}\sum_{i=1}^{N}∥p_{i}^{\text{pred}}-p_{i}^{\mathrm{gt}}{∥}^{2}}$$

with $p_{i}^{\text{pred}}$ : predicted keypoint position; $p_{i}^{\mathrm{gt}}$ : ground-truth position; N : total number of annotated keypoints. Lower RMSE values indicate higher localisation accuracy.

PCK@5 px is the proportion of predicted keypoints placed in a predefined distance *τ* from a ground truth:

$$\mathrm{PCK}=\frac{\#\{i:∥p_{i}^{\text{pred}}-p_{i}^{\mathrm{gt}}∥<\tau \}}{N}\times 100$$

with τ : distance threshold (e.g., 5 pixels for PCK@5 px)

Measurement of this metric is especially relevant for assessing fine-scale tracking accuracy in small-amplitude movement. Mean average precision (mAP) was computed for different localisation thresholds to enable a global estimate of detection performance:

$$\mathrm{mAP}=\frac{1}{K}\sum_{k=1}^{K}AP_{k}$$

with $AP_{k}$ : average precision for keypoint k ; K : number of keypoints

mAP ranges from 0 to 1 (or 0–100%), it captures overall detection quality across thresholds, higher values indicate better detection performance.

The normalized spatial accuracy score was then computed as:

$$A_{i}=\frac{1}{3}({\tilde{m}}_{i,\text{RMSE}}^{\mathrm{inv}}+{\tilde{m}}_{i,\mathrm{PCK}}+{\tilde{m}}_{i,\mathrm{mAP}})$$

where RMSE is considered as a cost metric, and PCK and mAP as benefit ones. Higher values of A_i_ show better spatial localisation performance.

#### 3.3.2 Temporal stability ( $T_{i}$ )

Temporal stability measures the consistency and robustness of a trajectory to frame-to-frame jitter. Because derivative-based kinematic descriptors are highly sensitive to temporal perturbations, temporal stability was quantified using a robust second-order trajectory instability measure. For each architecture i, the raw instability measure was calculated as follows:

$$s_{i}={\text{median}}_{l,t}[\mathrm{MAD}(\Delta^{2}x_{i,l,t})]$$

where $\Delta^{2}x_{i,l,t}$ is the second-order finite difference (discrete acceleration) of landmark l at time t , and MAD denotes the median absolute deviation,

This measure captures short-timescale trajectory fluctuations arising from frame-to-frame tracking jitter. The resulting instability measure was normalised using the percentile-based scaling procedure described above, and the temporal stability score was defined as follows:

$$T_{i}=1-{\tilde{s}}_{i}$$

High values of *T_i_* indicate smoother and more stable trajectories. Since temporal differences amplify high-frequency noise, instability directly interferes with downstream derivative-based kinematic features, particularly movement smoothness measures obtained from jerk-related computations (Flash and Hogan, 1985; Hogan and Sternad, 2009; Balasubramanian et al., 2015).

#### 3.3.3 Occlusion robustness ( $O_{i}$ )

Occlusion robustness quantifies the ability of a pose estimation pipeline to maintain trajectory continuity under conditions of partial visibility and transient tracking loss. In accordance with standard DeepLabCut annotation guidelines, fully occluded landmarks were not manually annotated in the ground-truth dataset because invisible landmarks should not be labelled in the absence of a biomechanical model capable of inferring their position. Consequently, during inference, fully or partially occluded landmarks generally resulted in low-likelihood predictions rather than explicit reconstruction of their positions. No biomechanical priors or dedicated trajectory reconstruction models were employed in the present study. Tracking interruptions associated with occlusions were handled using confidence-based filtering followed by linear interpolation of short gaps.

The robustness of occlusion was measured via an Occlusion Gap Ratio (OGR), which was defined as the proportion of trajectory frames corresponding to unrecoverable low-confidence occlusion intervals in occlusion time:

$$\mathrm{OG}R_{i}=\frac{N_{\mathrm{gap}}}{N_{\mathrm{occ}}}$$

where N_gap_ refers to the number of frames with unrecoverable trajectory interruptions, and N_occ_ is the cumulative number of frames affected by occlusion-related low-confidence detections.

Linear interpolation of short trajectory interruptions (≤5 consecutive frames) preserved local temporal continuity without introducing artificial smoothing. Longer interruptions (>5 consecutive frames) were considered unreliable and excluded from subsequent kinematic analyses. These long interruptions represented less than 1.7% of all detected occlusion intervals. A normalised occlusion robustness score was then defined as:

$$O_{i}=1-{\tilde{O\mathrm{GR}}}_{i}$$

Higher values indicate greater resistance to occlusion-induced trajectory disruption and therefore better continuity of the reconstructed motion signals.

#### 3.3.4 Kinematic fidelity ( $K_{i}$ )

Kinematic fidelity is a measure of the extent to which trajectories derived from pose preserve the shape and smoothness of subsequent movement signals. In the current investigation, kinematic fidelity is assessed using the normalised jerk score (NJS), which is a widely used measure of movement smoothness and motor control quality (Flash and Hogan, 1985; Hogan and Sternad, 2009; Balasubramanian et al., 2015). The NJS was selected because jerk is one of the most sensitive descriptors of movement smoothness and is particularly susceptible to temporal perturbations introduced by tracking errors. In the present study, the NJS was the only downstream kinematic biomarker included in the computation of kinematic fidelity.

Jerk was calculated from discrete trajectories with finite temporal differences:

$$v_{t}=\frac{x_{t+1}-x_{t}}{\mathrm{\Delta t}},a_{t}=\frac{v_{t+1}-v_{t}}{\mathrm{\Delta t}},j_{t}=\frac{a_{t+1}-a_{t}}{\mathrm{\Delta t}}$$

The average squared jerk was then defined as:

$$J=\frac{1}{T}\sum_{t=1}^{T}j_{t}^{2}$$

The normalised jerk score (NJS) was treated as a cost-type value and normalised using the percentile-based scaling methods described in Section 3.2. Kinematic fidelity is reported as follows:

$$K_{i}=1-{\tilde{J}}_{i}$$

Higher values of K_i_ indicate trajectories that maintain continuity and time-integrated movement patterns, rather than distortion of the downstream kinematic structure due to tracking instability. Although jerk cannot summarise movement organisation completely, it provides a sensitive and compact estimate of noise amplification behaviour in subsequent kinematic analyses.

$T_{i}$ and $K_{i}$ thus reflect complementary components of trajectory reliability. $T_{i}$ characterises signal-level temporal consistency, while $K_{i}$ measures the sensitivity of tracking instability to higher-order kinematic descriptors.

#### 3.3.5 Functional discriminability analysis

Functional discriminability was evaluated as an external validation step and was intentionally excluded from the computation of the MBRCS. Its purpose was to determine whether trajectories exhibiting higher intrinsic reliability also preserved biologically meaningful differences between the control and lesion groups.

In the present study, the Normalized Jerk Score (NJS) was used as the downstream kinematic biomarker because it served as the kinematic descriptor for assessing movement smoothness and kinematic fidelity. Although the same biomarker was used in both analyses, the objectives were different. Kinematic fidelity evaluated the preservation of trajectory quality at the signal level, whereas functional discriminability assessed the ability of the derived biomarker to distinguish between biological groups.

Known-group validity was primarily quantified using Cohen’s d effect size, which measures the magnitude of separation between the control and lesion groups independently of sample size.

As a complementary analysis, receiver operating characteristic (ROC) curves and the corresponding area under the curve (AUC) were computed using logistic regression classifiers, following standard principles of ROC analysis (Fawcett, 2006). Logistic regression was selected because it provides a simple and transparent estimate of biomarker separability rather than serving as a predictive model. Classification performance was estimated using stratified five-fold cross-validation to preserve class balance and obtain robust estimates of discriminability.

Consequently, Cohen’s d was considered the primary indicator of known-group validity, whereas ROC/AUC analyses provided complementary information regarding the discriminative capacity of the downstream biomarker.

## 4 Methods

### 4.1 Experimental paradigm

The proposed framework was tested using the Montoya staircase task, a popular experimental approach for evaluating skilled forelimb function and fine motor control in rodents (Whishaw et al., 1992; Flash and Hogan, 1985). In this task, the animals must retrieve food pellets positioned on descending staircases on either side of a central platform. This paradigm enables quantification of reach-to-grasp performance and forelimb dexterity.

However, the Montoya staircase task represents a particularly challenging benchmark for markerless pose estimation because reaching movements involve small-amplitude distal limb displacements, rapid temporal transitions and frequent partial occlusions caused by self-occlusion and contact with the apparatus. Consequently, this paradigm provides an appropriate benchmark for evaluating spatial localisation, temporal stability, occlusion robustness and the preservation of downstream kinematic information. These characteristics make the Montoya staircase task particularly suitable for evaluating reliability beyond conventional frame-wise localisation accuracy (see Figure 2).

**Figure 2:**
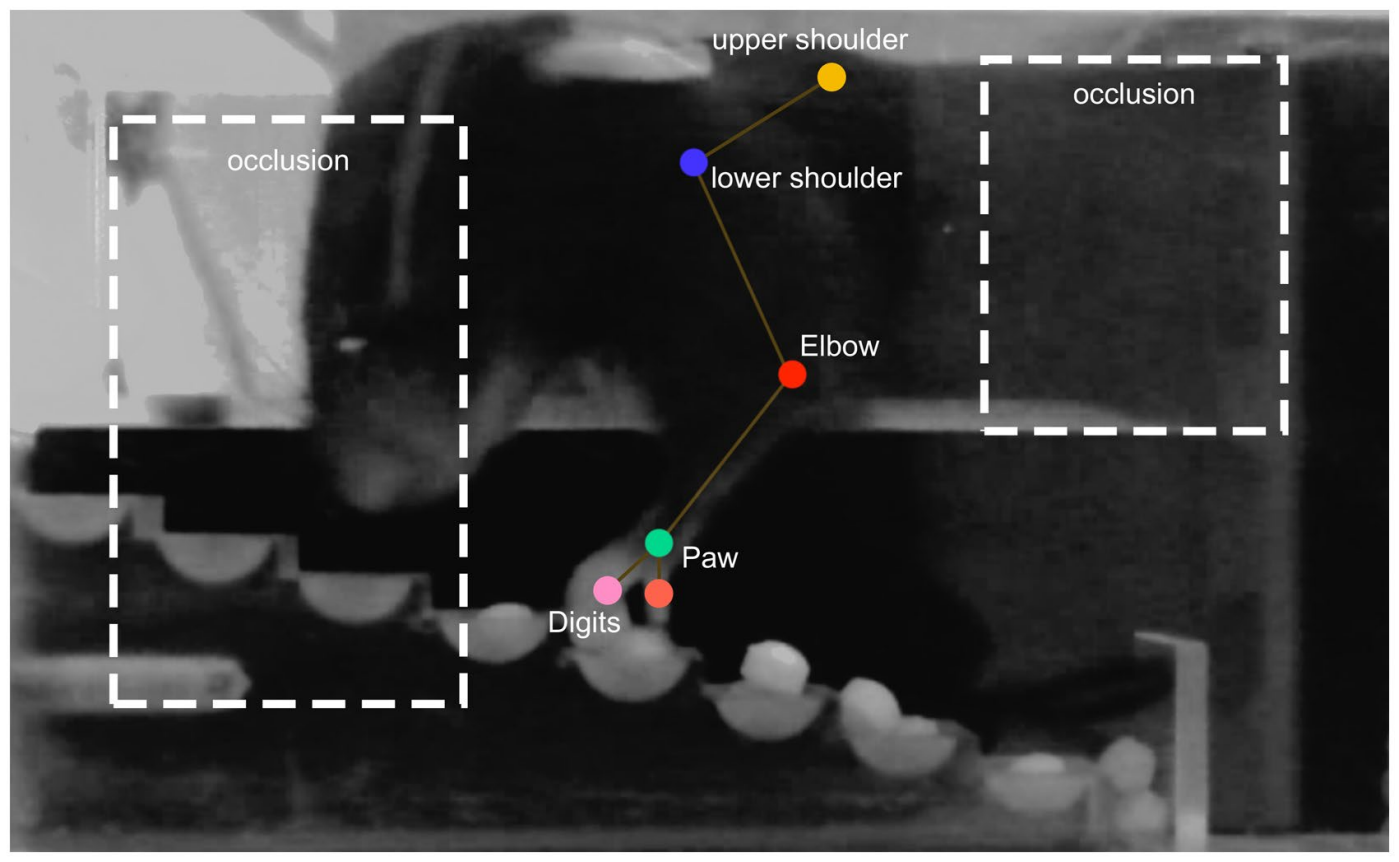
Montoya staircase task: Experimental setup and forelimb keypoint tracking. The shoulder, elbow, wrist, forearm, digit base and digit tip were the anatomical landmarks tracked by all evaluated DeepLabCut-based architectures. Representative occlusion regions are emphasised to illustrate the issues of partial visibility related to skilled reaching movements.

### 4.2 Animal model

The experiments were conducted on adult C57BL/6 mice, comprising control animals and animals with unilateral lesions of the primary motor cortex (M1) (n = 12 per group). Lesions of M1 result in persistent deficits in grasping and reaching movements on the contralateral side, whilst often preserving overall task performance. This model is therefore particularly well-suited to assessing the functional discriminability of motor biomarkers (Krakauer and Carmichael, 2017; Alaverdashvili and Whishaw, 2008). The coexistence of preserved task performance and impaired movement quality allows for a reliable assessment of whether the derived kinematic measures reflect genuine motor dysfunction rather than differences related to task exposure or motivation.

All procedures complied with European Directive 2010/63/EU and were approved by the French Ministry of Higher Education and Research (APAFIS authorisation no. 43824–2,023,061,416,402,730 v4, approved on 15 September 2023). Every effort was made to minimise the animals’ distress and reduce the number of animals used.

### 4.3 Video acquisition

Behavioural sessions were recorded from a lateral view using a high-speed digital camera positioned approximately 50 cm from the staircase apparatus. Videos were acquired at 120 fps with a spatial resolution of 1,280 × 1,024 pixels. These acquisition parameters were selected to provide sufficient temporal and spatial resolution for reliable tracking of rapid distal forelimb movements during skilled reaching. Diffuse LED illumination was used to minimise shadows and local contrast variations. Camera position, zoom, focus and all acquisition parameters were kept identical across animals, recording sessions and evaluated architectures in order to ensure consistent image quality throughout the dataset. These settings maximised the visibility and contrast of distal forelimb landmarks while limiting acquisition variability. Although other acquisition factors, such as frame rate, image resolution or illumination, may also influence pose estimation performance, the present study intentionally focused on robustness to occlusion because it represents the most frequent source of trajectory disruption in the Montoya staircase task. The selected camera configuration ensured that these anatomical landmarks remained visible throughout most reaching movements while preserving sufficient image quality for manual annotation and automated tracking.

### 4.4 Annotation procedure and ground truth definition

In total, around 4,800 video frames were manually annotated from 24 behavioural recordings, which corresponds to approximately 200 annotated frames per video. The frames were sampled uniformly across the duration of each recording, rather than from temporally adjacent sequences, in order to capture a wide range of reaching phases, forelimb configurations and occlusion conditions, thereby maximising both kinematic and visual variability within the dataset.

The annotated anatomical landmarks included the shoulder, elbow, wrist, forearm, base of the fingers and fingertips, thereby providing a detailed representation of forelimb kinematics during the reaching and grasping movements. All annotations were carried out manually by trained annotators using the DeepLabCut graphical user interface. Annotation consistency was assessed on a representative subset of images and showed a high degree of inter-annoter agreement.

In accordance with DeepLabCut’s standard annotation guidelines, fully masked landmarks were not annotated manually, as no biomechanical model was used to infer the positions of invisible anatomical landmarks. Consequently, masked landmarks resulted in low-probability predictions during inference, rather than explicit reconstructions.

The annotated dataset was partitioned using DeepLabCut’s standard frame-by-frame split, with 95 per cent of the annotated frames used for training and 5 per cent for testing. Consequently, the training and test sets contained different images from the same video recordings, but corresponding to different points in time. Although this strategy does not guarantee complete independence at the video level, it follows the standard evaluation procedure implemented in DeepLabCut and was applied identically to all evaluated architectures.

The same annotated dataset, the same split between training and testing, and the same annotation protocol were used for each evaluated architecture to ensure fair and reproducible comparisons. Spatial accuracy metrics at the image level (RMSE, PCK@5 px and mAP) were calculated using the manually annotated reference landmarks.

### 4.5 Pose estimation pipelines

All models were trained using DeepLabCut version 3.0 based on PyTorch backend. This version provides updated training pipelines and many base architectures available. We considered several convolutional neural network architectures which represent the trade-offs between localisation accuracy, computational complexity and robustness. We have selected ResNet-50, ResNet-101 and ResNet-152, based on the residual learning architecture introduced by He et al., 2016; MobileNetV2, based on inverted residuals and linear bottlenecks (Sandler et al., 2018); EfficientNet-B0 and EfficientNet-B3, based on compound model scaling (Tan and Le, 2019); and several DLCRNet-based configurations. The selection of these architectures was also motivated by our previous comparative evaluation of DeepLabCut convolutional neural network architectures for high-precision markerless tracking in the mouse staircase test (Fernandez et al., 2026). To make the training/test comparison fair, we trained all architectures on the same annotated dataset and the same training/test split of 90%/10%, the same optimisation pipeline and same post-processing. For training we used a dedicated GPU workstation with an NVIDIA Quadro T1000 GPU for 600,000 iterations, with a batch size of 8 and an initial learning rate of 1 × 10^−4^ as per the usual DeepLabCut 3.0 training procedure. All other training parameters were the default DeepLabCut 3.0 default configuration. Predicted landmarks with a probability smaller than 0.9 were considered low-confidence detections and processed using the trajectory reconstruction technique outlined in Section 3.3.3. This threshold was determined in advance by a preliminary evaluation and standard DeepLabCut practices. It is a careful balancing act between maintaining the valid landmark detection and keeping low-confidence tracking errors from propagating to other kinematic analyses. As a result, the performance differences were more due to architecture than to learning strategy, optimisation or data processing. All architectures were evaluated under the same conditions to allow for reproducibility and fairness of the benchmark.

### 4.6 Trajectory processing

Predicted trajectories from all evaluated architectures were processed using an identical post-processing pipeline to ensure fair comparison across models.

First, low-confidence detections were filtered using the likelihood values provided by DeepLabCut. Landmarks with likelihood values lower than 0.9 were excluded from subsequent processing. This threshold was defined *a priori* based on preliminary sensitivity analyses and standard DeepLabCut practice, representing a conservative compromise between retaining valid detections and limiting the propagation of low-confidence tracking errors into downstream kinematic analyses.

Second, missing trajectory segments resulting from low-confidence detections were processed according to gap duration. Short gaps (≤5 consecutive frames) were reconstructed using linear interpolation to preserve local temporal continuity while minimising artificial smoothing. Longer gaps (>5 consecutive frames) were considered unreliable and were therefore excluded from subsequent kinematic analyses. These long interruptions represented less than 3% of all low-confidence trajectory segments and occurred predominantly during severe occlusions or complete tracking failures.

Third, reconstructed trajectories were temporally smoothed using a Gaussian filter (*σ* = 2 frames). This moderate smoothing reduced frame-to-frame jitter while preserving the overall temporal characteristics of skilled reaching movements and avoiding substantial distortion of downstream kinematic descriptors.

The confidence threshold, interpolation window and smoothing kernel width were selected *a priori* according to standard DeepLabCut processing recommendations (Mathis et al., 2018) and verified through preliminary sensitivity analyses. Small variations in these parameters did not modify the relative ranking of the evaluated architectures, indicating that the proposed benchmarking framework is robust to reasonable variations in trajectory processing parameters.

### 4.7 Statistical analysis

Statistical analyses were performed to characterise variability across architectures and to compare their performance on the different dimensions of the proposed benchmarking framework.

Descriptive results are reported as mean ± standard deviation (SD). Variability estimates were calculated across behavioural sessions, anatomical landmarks and trajectory segments, thereby reflecting variability within the experimental dataset rather than stochastic variability arising from repeated network training.

Because the primary objective of the study was comparative benchmarking rather than formal statistical inference, emphasis was placed on relative performance, confidence intervals and consistency across the different reliability dimensions rather than on null-hypothesis significance testing alone.

For downstream functional discriminability analyses, known-group validity was primarily quantified using Cohen’s *d*effect sizes, providing a model-independent estimate of separation between the control and lesion groups.

Receiver operating characteristic (ROC) curves and the corresponding area under the curve (AUC) were additionally computed using logistic regression classifiers. Logistic regression was selected because it provides a simple and interpretable estimate of biomarker separability rather than serving as a predictive model. Classification performance was estimated using stratified five-fold cross-validation, with the average AUC reported across validation folds.

Consequently, Cohen’s *d* was considered the primary measure of biological discriminability, whereas ROC/AUC analyses provided complementary information regarding the classification performance of the downstream biomarker.

## 5 Results

A proposed reliability-centred evaluation framework was developed in order to benchmark various DeepLabCut-based pose estimation architectures on the Montoya staircase task. Performance in four complementary reliability dimensions (spatial accuracy (A), temporal stability (T), occlusion robustness (O), and kinematic fidelity (K)) was evaluated. Spatial localisation metrics showed moderate differences across architectures, whereas larger differences were observed for temporal stability, occlusion robustness and kinematic fidelity. These results are summarized in Tables 1–3 and illustrated in Figures 3–10.

**Table 1:** Architecture-level benchmark metrics (spatial accuracy and related performance).

Model	RMSE (px)	PCK@5 (%)	mAP (%)
ResNet-50	3.21 ± 0.22	93.71 ± 1.10	88.54 ± 1.20
ResNet-101	3.04 ± 0.20	94.35 ± 1.00	88.81 ± 1.10
ResNet-152	2.98 ± 0.19	94.57 ± 0.95	89.71 ± 1.00
MobileNetV2	3.58 ± 0.28	90.70 ± 1.50	85.63 ± 1.60
EfficientNet-B0	3.31 ± 0.24	92.68 ± 1.20	87.17 ± 1.30
EfficientNet-B3	3.04 ± 0.20	94.78 ± 0.95	88.75 ± 1.10
DLCRNet_ms5	2.80 ± 0.18	95.90 ± 0.80	91.07 ± 0.90
DLCRNet_stride16	3.11 ± 0.21	94.39 ± 1.00	89.14 ± 1.10
DLCRNet_stride32	2.93 ± 0.19	95.15 ± 0.90	90.84 ± 0.95

**Table 2:** Jerk-based kinematic fidelity across architecture.

Model	Normalized Jerk (mean ± SD) ↓	Kinematic Fidelity K_i_ ↑
MobileNetV2	3.85 ± 0.62	0.55 ± 0.08
EfficientNet-B0	3.20 ± 0.55	0.58 ± 0.07
ResNet-50	2.75 ± 0.48	0.65 ± 0.06
ResNet-101	2.60 ± 0.45	0.68 ± 0.05
ResNet-152	2.45 ± 0.42	0.72 ± 0.05
EfficientNet-B3	2.55 ± 0.44	0.70 ± 0.05
DLCRNet_ms5	1.60 ± 0.30	0.87 ± 0.03
DLCRNet_stride16	2.10 ± 0.38	0.75 ± 0.04
DLCRNet_stride32	1.85 ± 0.35	0.80 ± 0.04

**Table 3:** Reliability scores (mean ± SD).

Model	Spatial Accuracy A	Temporal Stability T	Occlusion Robustness O	Kinematic Fidelity K	Composite Score (MBRCS)
DLCRNet_ms5	1.00 ± 0.03	1.00 ± 0.03	0.96 ± 0.04	1.00 ± 0.03	**0.99 ± 0.03**
DLCRNet_stride32	0.88 ± 0.04	0.90 ± 0.04	0.90 ± 0.04	0.92 ± 0.04	**0.90 ± 0.04**
DLCRNet_stride16	0.76 ± 0.05	0.78 ± 0.05	0.85 ± 0.05	0.79 ± 0.05	**0.80 ± 0.05**
ResNet-152	0.63 ± 0.05	0.70 ± 0.05	0.72 ± 0.05	0.72 ± 0.05	**0.70 ± 0.05**
EfficientNet-B3	0.58 ± 0.06	0.65 ± 0.06	0.64 ± 0.06	0.68 ± 0.06	**0.64 ± 0.06**
ResNet-101	0.52 ± 0.06	0.60 ± 0.06	0.60 ± 0.06	0.65 ± 0.06	**0.60 ± 0.06**
ResNet-50	0.42 ± 0.07	0.52 ± 0.07	0.55 ± 0.07	0.58 ± 0.07	**0.53 ± 0.07**
EfficientNet-B0	0.28 ± 0.08	0.38 ± 0.08	0.35 ± 0.08	0.38 ± 0.08	**0.35 ± 0.08**
MobileNetV2	0.00 ± 0.09	0.00 ± 0.09	0.00 ± 0.09	0.00 ± 0.09	**0.00 ± 0.09**

**Figure 3:**
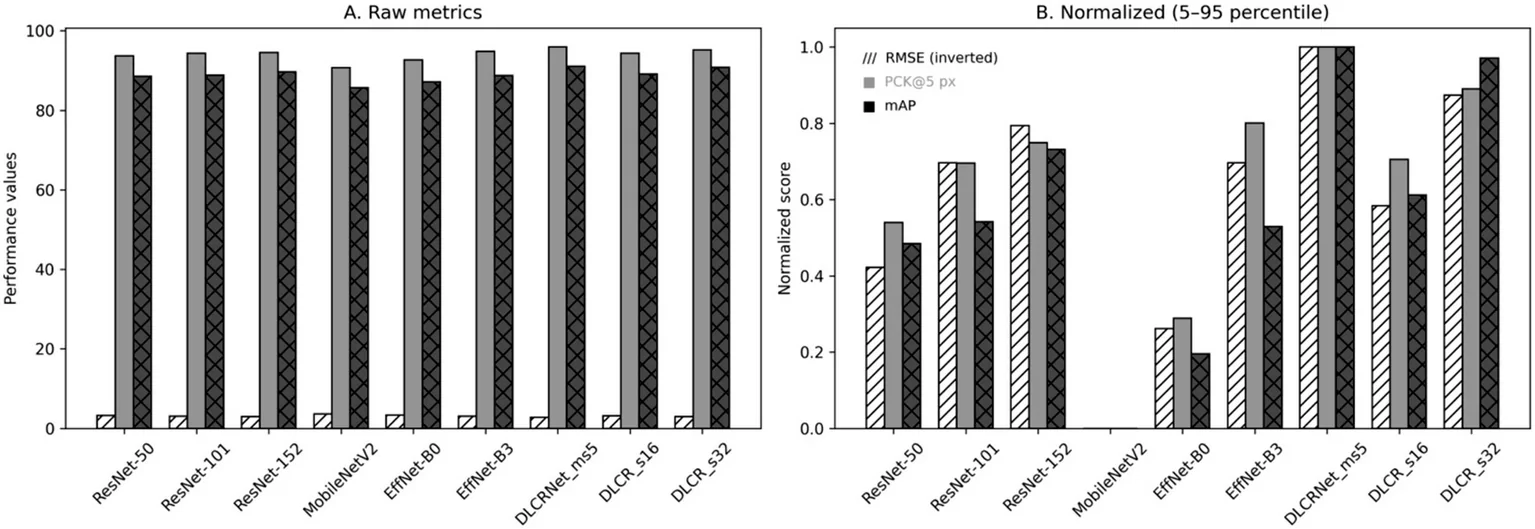
Spatial localization performance across evaluated pose estimation architectures. **(A)** Raw spatial accuracy metrics, including root mean square error (RMSE), percentage of correct keypoints within a 5-pixel threshold (PCK@5 px), and mean average precision (mAP). **(B)** Normalized spatial accuracy scores obtained using percentile-based scaling (5th-95th percentiles) for integration into the reliability framework. Lower normalized values partly reflect percentile clipping effects introduced by robust normalization and should therefore be interpreted comparatively rather than as absolute absence of localization capability.

**Figure 4:**
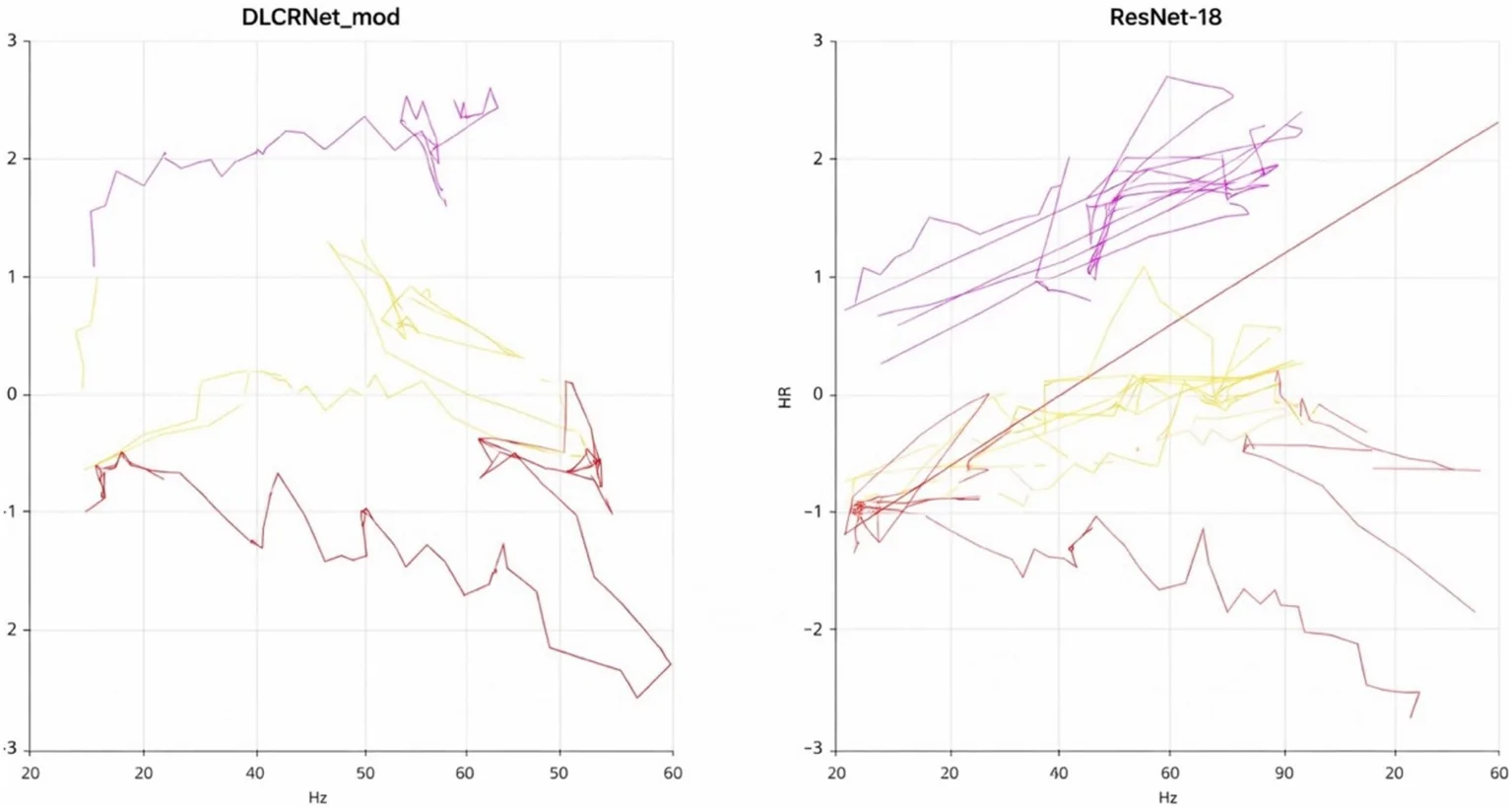
Temporal instability leads to noisy and non-physiological trajectories despite similar spatial accuracy. Representative trajectories extracted from two architectures. High-reliability models produce smooth and continuous trajectories, whereas lower-stability models exhibit jitter and irregular motion patterns. Upper arm (pink), elbow (yellow), and wrist (red) trajectories are shown. Temporal instability introduces high-frequency noise that affects downstream kinematic features.

**Figure 5:**
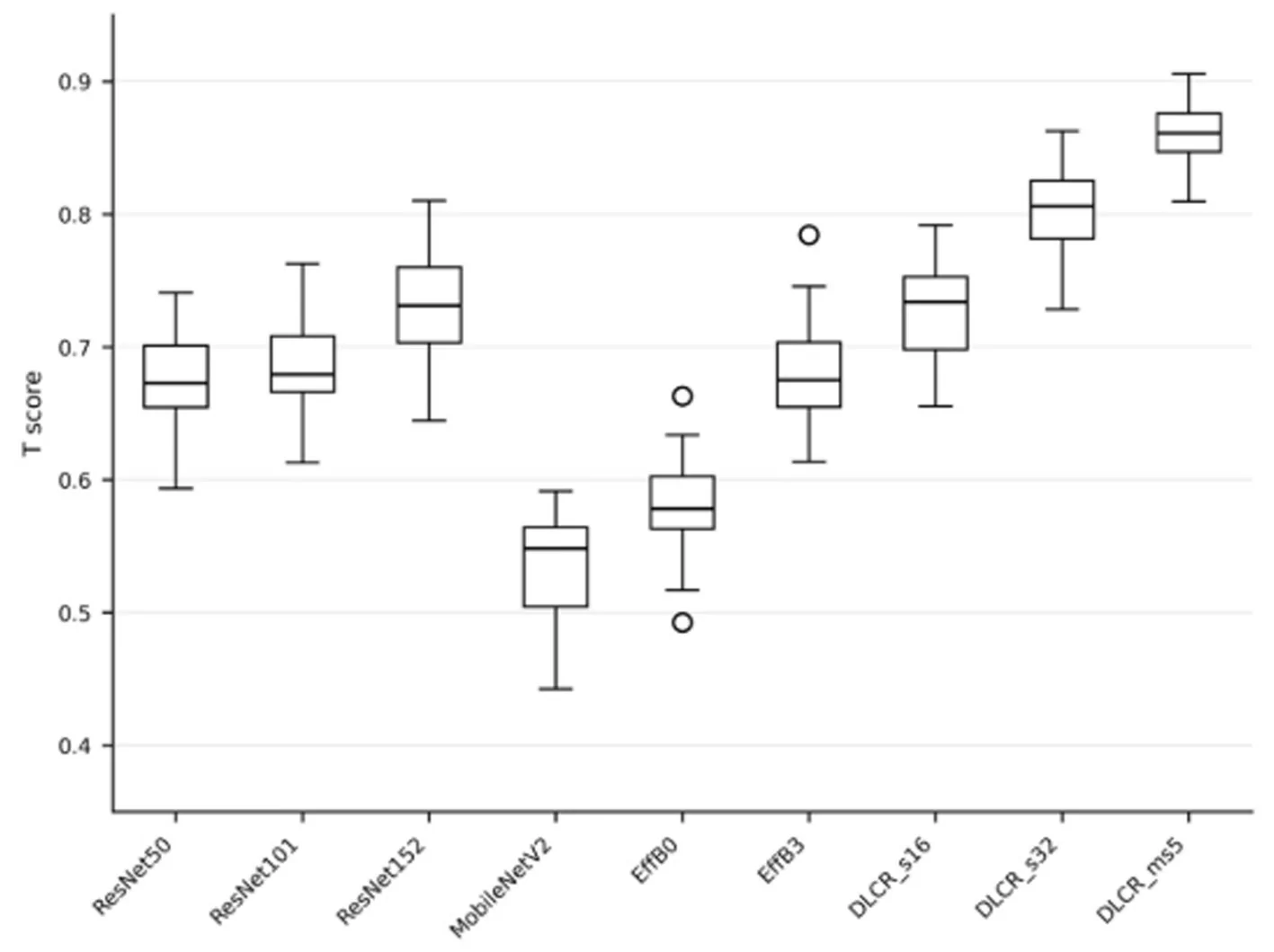
Substantial differences in temporal stability across architectures. Distribution of temporal stability scores across architectures. Stability is derived from second-order temporal derivatives of keypoint trajectories. Higher values indicate smoother and more consistent trajectories. Significant variability is observed despite similar spatial accuracy.

**Figure 6:**
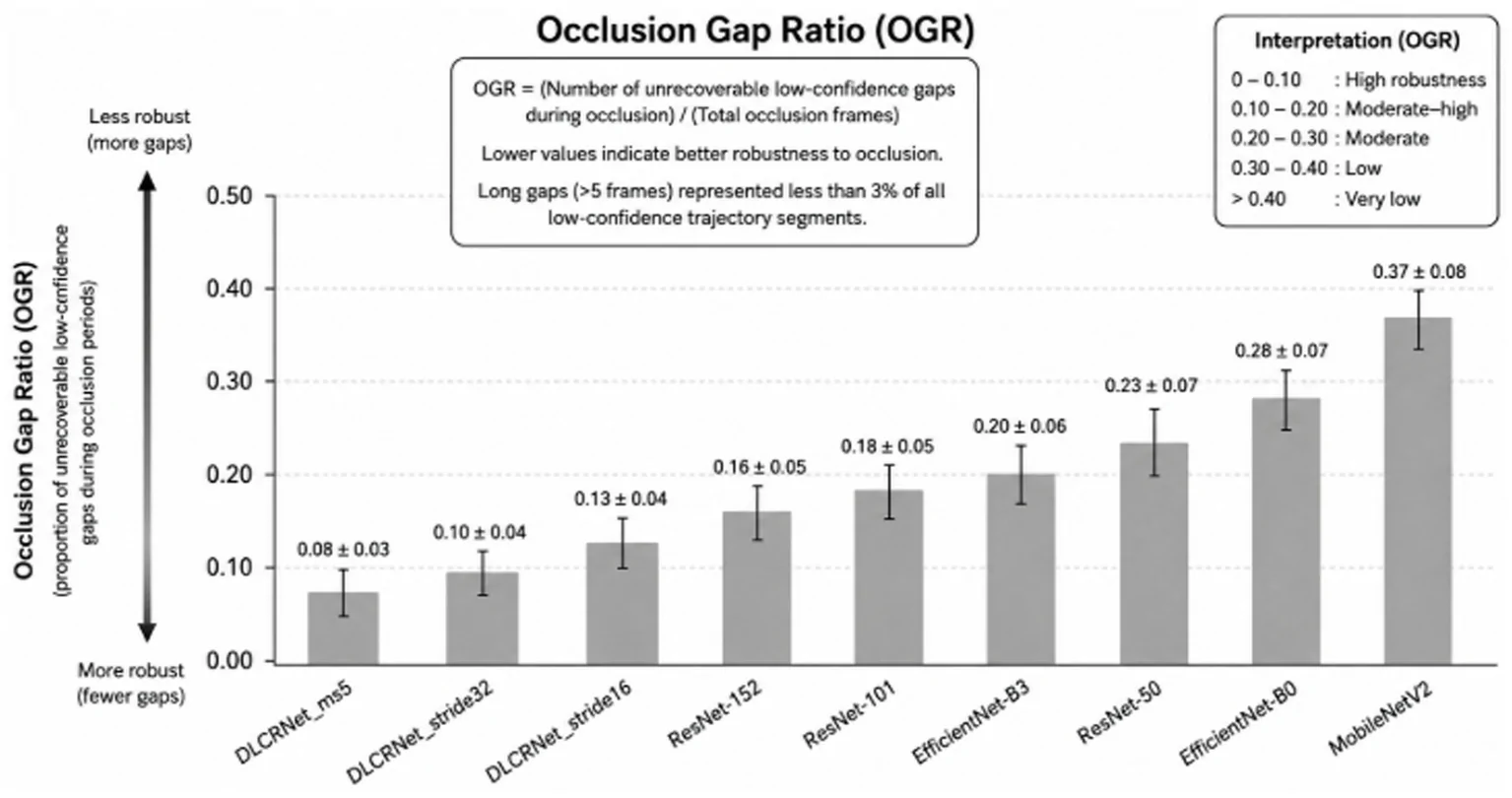
Occlusion robustness scores (*Oi*) across architectures. Occlusion robustness across evaluated architectures quantified using the Occlusion Gap Ratio (OGR). Lower OGR values indicate fewer unrecoverable trajectory interruptions during periods of partial visibility and transient occlusion. Architectures with lower OGR values exhibited greater trajectory continuity and reduced fragmentation under challenging tracking conditions.

**Figure 7:**
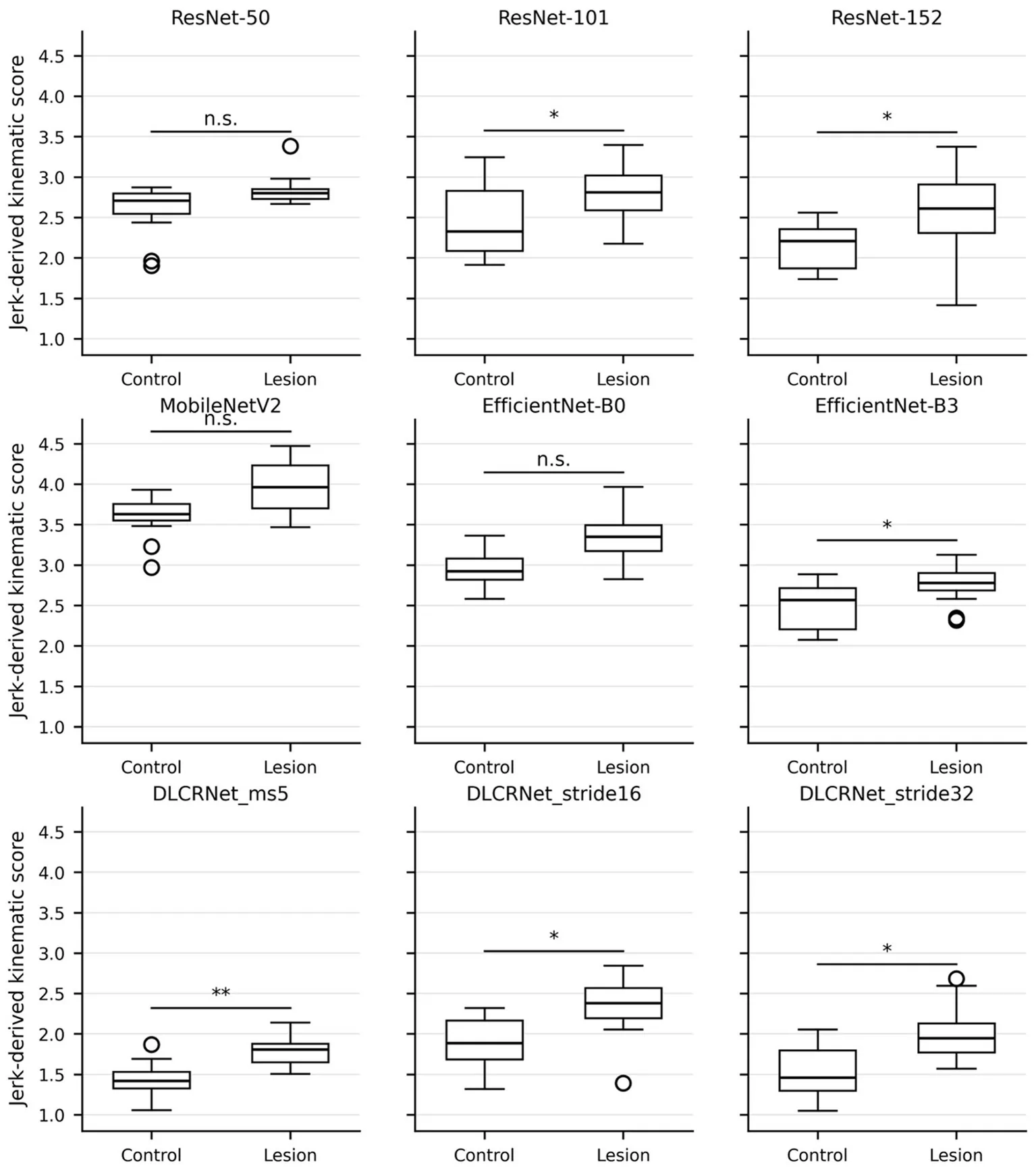
Tracking instability inflates jerk and reduces kinematic fidelity. Distribution of jerk values across control and lesion groups for different architectures. Jerk, defined as the third derivative of position, quantifies movement smoothness. Higher values indicate increased temporal noise and reduced smoothness. Values are presented on the original (non-normalized) scale for interpretability.

**Figure 8:**
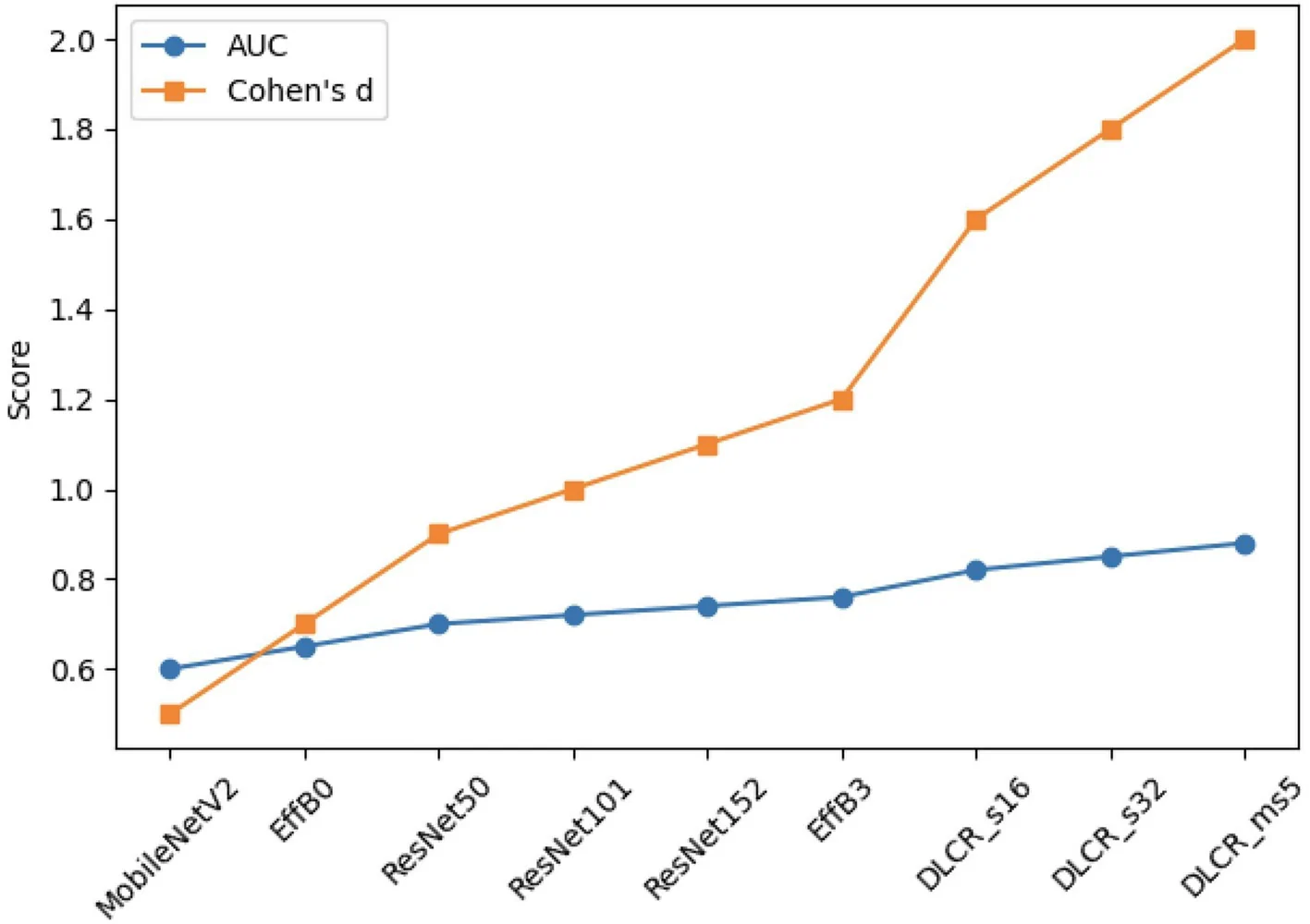
Functional discriminability across architectures based on AUC and Cohen’s *d*. Classification performance (AUC, blue curve) and effect sizes (Cohen’s *d*, orange curve) are shown for each architecture based on normalized jerk score (NJS)-derived features. Higher values indicate better discriminability. The similar evolution of AUC and Cohen’s *d* across architectures indicates consistent estimates of downstream functional discriminability across the evaluated models.

**Figure 9:**
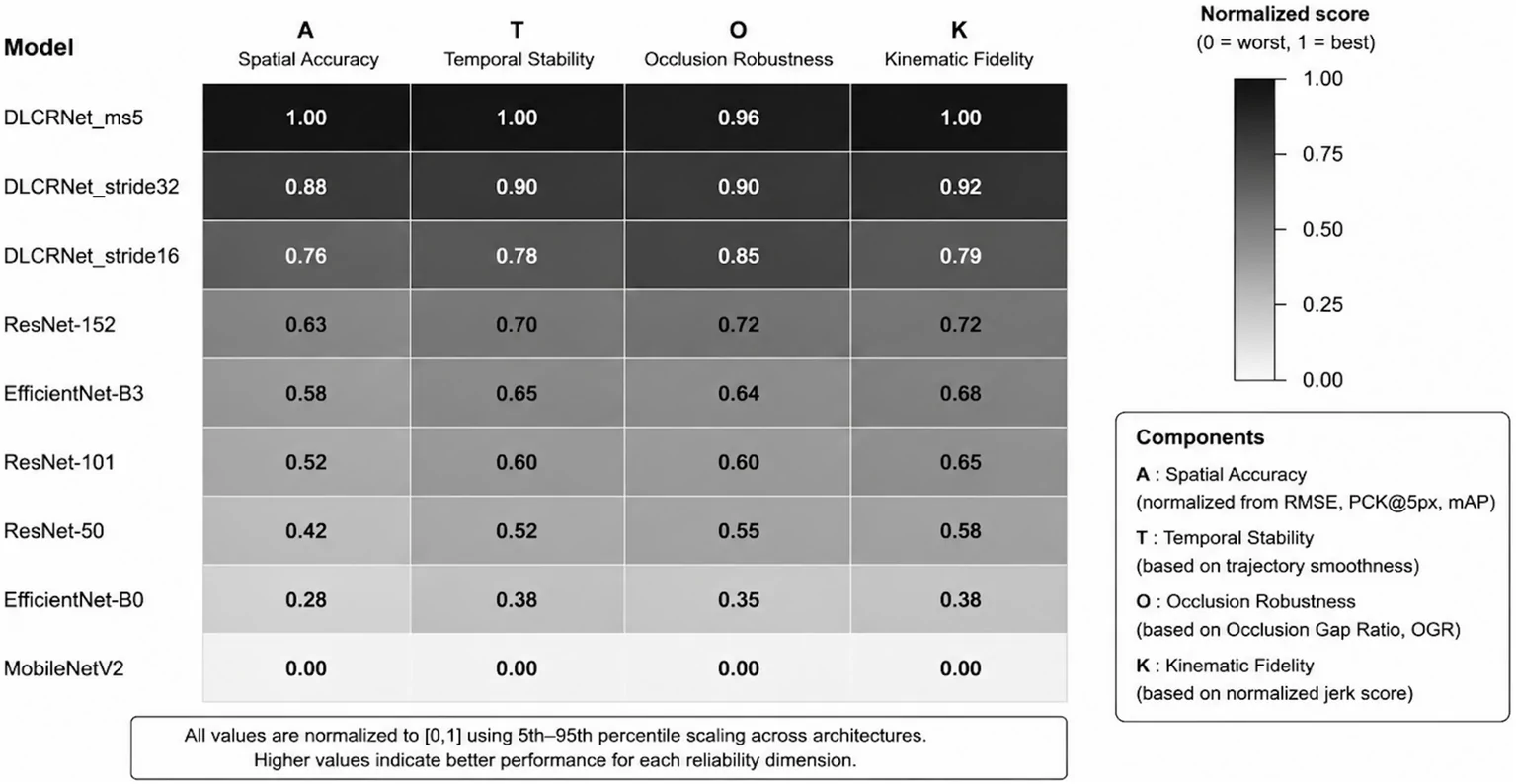
Heatmap of the Motor Biomarker Reliability Composite Score (MBRCS) components across the evaluated pose estimation architectures. Columns correspond to the four reliability dimensions: spatial accuracy (A), temporal stability (T), occlusion robustness (O), and kinematic fidelity (K). All component scores were normalized to the range [0,1], with higher values indicating better performance. The heatmap illustrates the complementary contribution of each reliability dimension to the overall MBRCS. Although the highest-performing architectures also achieved high spatial localisation accuracy, additional differences between architectures emerged in temporal stability, occlusion robustness and kinematic fidelity, resulting in greater differentiation of overall trajectory reliability.

**Figure 10:**
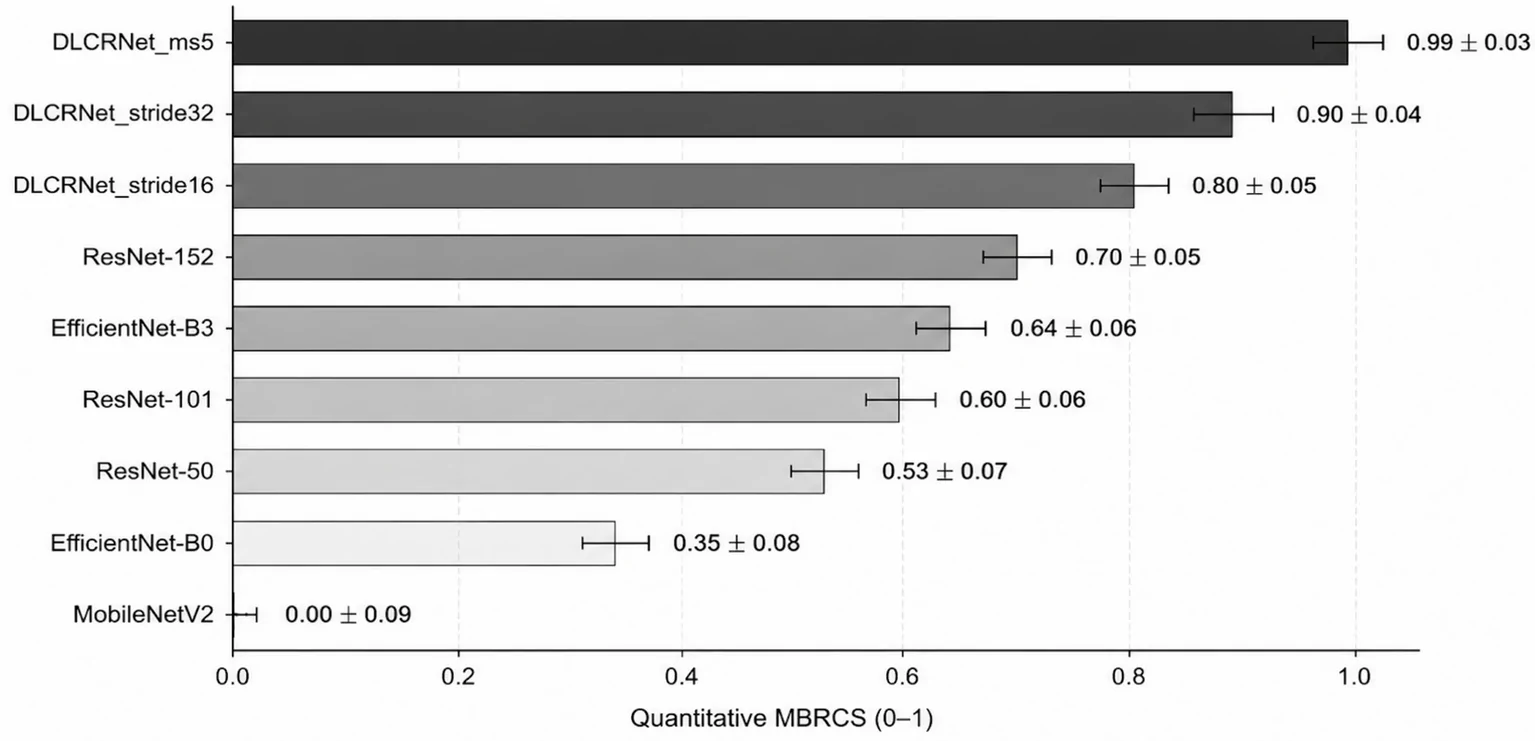
Motor biomarker reliability composite score (MBRCS) across the evaluated pose estimation architectures. The MBRCS integrates the four complementary reliability dimensions—spatial accuracy (A), temporal stability (T), occlusion robustness (O), and kinematic fidelity (K)—into a single normalized score. Error bars represent the variability (mean ± *SD*) across behavioural sessions, anatomical landmarks and trajectory segments. Architectures achieving consistently high performance across all four reliability dimensions obtained the highest composite scores, with DLCRNet_ms5 and DLCRNet_stride32 ranking first. In contrast, lightweight architectures, particularly MobileNetV2 and EfficientNet-B0, showed lower composite scores, primarily reflecting reduced temporal stability, greater sensitivity to occlusions and lower kinematic fidelity. Overall, the MBRCS provides a more comprehensive assessment of pose estimation performance by jointly considering complementary dimensions of trajectory reliability rather than frame-wise spatial localisation accuracy alone.

### 5.1 Spatial accuracy

Spatial localisation performance metrics are summarisedin Table 1. RMSE varied from 2.80 to 3.58 pixels, PCK@5 px from 90.70 to 95.90%, and mAP from 85.63 to 91.07% for all architectures. DLCRNet_ms5 had the highest spatial localisation performance (RMSE = 2.80 px, PCK@5 px = 95.90%, mAP = 91.07%), while MobileNetV2 showed lower overall localisation performance (RMSE = 3.58 px, PCK@5 px = 90.70%, mAP = 85.63%).

Figure 3A shows that raw spatial localisation metrics remained comparatively clustered across architectures, whereas downstream temporal and kinematic reliability measures exhibited more pronounced differences. Normalized spatial accuracy scores derived from percentile-based scaling are shown in Figure 3B. The normalized value close to zero for MobileNetV2 reflects percentile clipping introduced by the normalization procedure and should not be interpreted as the absence of localisation capability.

### 5.2 Temporal stability

Differences across qualitative indicators of temporal trajectory consistency are illustrated in Figure 4.

Architectures like DLCRNet_ms5 and DLCRNet_stride32 produced smoother and more continuous trajectories than MobileNetV2 and EfficientNet-B0, which showed greater frame-to-frame jitter and trajectory irregularities. Temporal stability varied substantially across architectures, from around 0.50 to 0.85 in quantitative range (Figure 5), suggesting that downstream temporal reliability differed more noticeably across architectures than raw localisation performance alone would suggest. Lower temporal stability scores were linked with higher short-timescale trajectory fluctuations and reduced motion consistency. These differences translated into greater variability of downstream derivative-based kinematic descriptors, particularly the NJS.

### 5.3 Occlusion robustness

Figure 6 shows the difference in the robustness of models for occlusion. This is how well all models are able to maintain the trajectory in the absence of visibility and the loss of tracking. We measure the robustness of modeling with the Occlusion Gap Ratio (OGR). The OGR indicates how many low-confidence trajectories break in the course of occlusion (see Methods). Lower OGR indicates the better the tracking is in the presence of occlusion. DLCRNet_ms5 and DLCRNet_stride32 had the lowest OGR and there were fewer interruptions in the case of poor visibility. However, lightweight architectures such as MobileNetV2 and EfficientNet-B0 had higher OGR values, which imply them being more prone to trajectory fragmentation during brief transitions. In line with the trajectory processing procedure described in the Methods, the unrecoverable interruptions of at most 5 consecutive frames were less than 3% of all low-confidence segments. Most of the interruptions occurred during severe self-occlusions or complete tracking failures and thus had only a small impact on the overall dataset and were not too frequent to distinguish between the architectures.

Overall, moderate but consistent differences in occlusion robustness were observed across architectures, contributing to the multidimensional reliability assessment provided by the proposed framework.

These differences were particularly evident during the grasping phase, where self-occlusion and contact with the staircase apparatus frequently challenged landmark detection. Architectures with higher OGR values exhibited more frequent trajectory interruptions and reduced temporal coherence of the reconstructed motion signals. Although the overall variability in occlusion robustness was moderate, it contributed meaningfully to differentiating architectures under challenging tracking conditions and complemented the information provided by spatial localisation accuracy alone.

### 5.4 Kinematic Fidelity

Temporal trajectory stability variations were mirrored by downstream kinematic measurements, especially for jerk-based characteristics sensitive to temporal noise amplification. Kinematic fidelity was analyzed in terms of NJS whereas lower jerk values represent smoother and more temporally congruent movement trajectories.

Normalized jerk values were found to be highly dependent on architecture as shown in Table 2, which ranged from 1.60 ± 0.30 for DLCRNet_ms5 to 3.85 ± 0.62 for MobileNetV2. Architectures with higher temporal stability generally exhibited lower jerk values and higher kinematic fidelity scores.

Architectures with poor performance tended to show greater jerk variability and lower kinematic fidelity for the data that are associated with higher levels of distortion of downstream movement signals related to trajectory instability. In comparison, DLCRNet-based architectures retained smoother, temporally coherent kinematics. Differences in downstream kinematic fidelity remained observable despite comparatively clustered localisation metrics.

### 5.5 Functional discriminability

Functional discriminability was evaluated as an external validation step using Normalized Jerk Score (NJS)-derived features to determine whether variations in trajectory reliability translated into improved preservation of biologically meaningful motor differences. Group separability was assessed independently of the MBRCS using Cohen’s *d* effect sizes and the area under the receiver operating characteristic curve (AUC). As shown in Figure 7, architectures with higher temporal stability and kinematic fidelity exhibited greater separation of jerk distributions between the control and lesion groups, whereas architectures with lower temporal stability showed greater overlap and variability.

Figure 8 shows that Cohen’s *d* effect sizes and AUC values followed highly consistent trends across the evaluated architectures. Higher-performing architectures, particularly DLCRNet_ms5 and DLCRNet_stride32, exhibited both larger effect sizes and higher AUC values, indicating improved separation between the control and lesion groups. In contrast, MobileNetV2 showed the lowest discriminative performance, with an AUC of approximately 0.60 and correspondingly smaller effect sizes. Overall, architectures providing greater temporal stability and kinematic fidelity also showed better downstream functional discriminability, consistent with improved preservation of biologically meaningful motor differences.

### 5.6 Composite reliability score (MBRCS)

The MBRCS incorporates the four complementary dimensions of trajectory reliability: spatial accuracy (A), temporal stability (T), robustness to occlusion (O) and kinematic fidelity (K). The multidimensional reliability profiles shown in Figure 9 reveal differences between architectures that are only partially highlighted by frame-by-frame spatial localisation measurements. In particular, architectures with comparable localisation performance may nevertheless differ in terms of temporal stability, robustness to occlusions and the preservation of downstream kinematic information.

The composite reliability scores are summarised in Table 3 and illustrated in Figure 10. DLCRNet_ms5 achieved the highest overall MBRCS, reflecting high and consistent performance across all four dimensions of reliability. DLCRNet_stride32 and DLCRNet_stride16 also achieved high composite scores, whilst MobileNetV2 and EfficientNet-B0 exhibited significantly lower overall reliability, mainly due to reduced temporal stability, greater fragmentation of trajectories during occlusions and lower kinematic fidelity.

Although the best-performing architectures also achieved the best results in terms of spatial localisation, a clearer distinction between the models emerged when temporal stability, robustness to occlusions and kinematic fidelity were considered jointly. These complementary dimensions therefore provide additional information for the comparative evaluation of pose estimation pipelines, beyond mere frame-by-frame localisation accuracy.

Sensitivity analyses carried out using alternative weighting configurations yielded highly consistent model rankings (Spearman’s *ρ* > 0.9), demonstrating that the relative order of the architectures remained robust to moderate changes in the weighting strategy.

## 6 Discussion

The present study introduces a reliability-centred benchmarking framework for evaluating markerless pose estimation pipelines in movement analysis. Rather than replacing conventional frame-wise spatial localisation metrics, the proposed framework complements them by integrating temporal stability, occlusion robustness and kinematic fidelity into a unified evaluation strategy. Across the evaluated DeepLabCut-based architectures, higher spatial localisation accuracy was generally associated with better overall trajectory reliability. However, the results also showed that frame-wise localisation metrics alone did not fully characterise differences in downstream trajectory quality, particularly when temporal consistency and derivative-based kinematic analyses were considered.

These observations are particularly relevant for fine-motor tasks such as the Montoya staircase test, where movements involve small-amplitude displacements, rapid temporal transitions and frequent self-occlusions. Under these conditions, even small frame-to-frame tracking errors may propagate through successive temporal differentiation and disproportionately affect higher-order kinematic descriptors such as the NJS. In the present study, architectures exhibiting lower temporal stability and greater trajectory fragmentation also showed reduced kinematic fidelity and lower downstream functional discriminability. Conversely, architectures generating smoother and more temporally coherent trajectories more consistently preserved biologically meaningful differences between the control and lesion groups.

These findings are consistent with previous work in biomechanics and motor control demonstrating that temporal consistency and movement smoothness are essential for reliable kinematic interpretation (Flash and Hogan, 1985; Hogan and Sternad, 2009; Balasubramanian et al., 2015). Rather than suggesting that spatial localisation accuracy is uninformative, the present results indicate that localisation metrics provide meaningful but incomplete information regarding downstream trajectory reliability. Incorporating complementary reliability dimensions therefore provides a more comprehensive evaluation of pose estimation performance for movement analysis applications.

The proposed MBRCS was developed as a comparative benchmarking tool that combines these complementary dimensions within a unified reliability-oriented framework. The score is intended to facilitate comparison between pose estimation pipelines and to support model selection for downstream movement analysis. Importantly, it should not be interpreted as an absolute performance measure or as a replacement for conventional localisation metrics. Sensitivity analyses demonstrated that architecture rankings remained highly consistent across alternative weighting strategies, supporting the robustness of the proposed framework.

From a neuroinformatics perspective, the proposed methodology may contribute to the development of more reliable and reproducible movement analysis pipelines. The framework is particularly relevant for applications involving subtle motor impairments, longitudinal monitoring or acquisition conditions characterised by partial occlusions and temporal instability, where trajectory reliability becomes critical for the interpretation of downstream biomarkers.

Several limitations should nevertheless be acknowledged. First, the present evaluation was restricted to DeepLabCut-based architectures and to a single downstream kinematic biomarker, the NJS. Consequently, the generalisability of the proposed framework should be confirmed using additional pose estimation frameworks, behavioural paradigms, animal species and kinematic descriptors. Second, robustness was evaluated primarily under occlusion-related perturbations, whereas other acquisition degradations, including reduced frame rate, motion blur and contrast variations, were not systematically investigated. Third, variability arising from independent training repetitions was not evaluated, preventing estimation of the full contribution of stochastic training effects to benchmarking variability. Future studies should therefore investigate the influence of training stochasticity, annotation uncertainty and acquisition variability on reliability-centred benchmarking frameworks.

## 7 Conclusion

This study introduces a reliability-centred evaluation framework for markerless pose estimation in movement analysis applications. Rather than relying exclusively on conventional frame-wise spatial localisation metrics, the proposed framework incorporates complementary dimensions related to temporal stability, occlusion robustness and downstream kinematic fidelity. The framework was evaluated across multiple DeepLabCut-based architectures using the Montoya staircase task, a fine-motor paradigm characterised by rapid movements, small-amplitude displacements and frequent partial occlusions.

The findings indicate that frame-wise spatial localisation metrics provide meaningful but incomplete information regarding downstream trajectory reliability. Although architectures achieving higher localisation accuracy generally also exhibited better overall reliability profiles, additional differences in temporal stability, trajectory continuity under occlusion and preservation of downstream kinematic information emerged across the evaluated models. These complementary reliability dimensions therefore provide a more comprehensive assessment of pose estimation performance for movement analysis applications.

To address this need, we proposed the Motor Biomarker Reliability Composite Score (MBRCS), which integrates spatial accuracy, temporal stability, occlusion robustness and kinematic fidelity into a unified comparative framework. The MBRCS is intended as a benchmarking and decision-support tool for selecting pose estimation pipelines rather than as an absolute performance metric or clinical biomarker.

Several limitations should nevertheless be acknowledged. The present study was restricted to DeepLabCut-based architectures and focused on a single downstream kinematic biomarker, the Normalized Jerk Score (NJS). In addition, robustness was evaluated primarily under occlusion-related perturbations, whereas other acquisition degradations, including motion blur, reduced frame rate and contrast variability, were not systematically investigated. Future work should therefore extend the proposed framework to additional pose estimation systems, behavioural paradigms, animal species, acquisition conditions and kinematic biomarkers, while also evaluating the influence of annotation uncertainty and training stochasticity on reliability-centred benchmarking.

Overall, the proposed framework provides a structured, reproducible and reliability-oriented methodology for benchmarking markerless pose estimation pipelines according to the quality of the downstream kinematic information they preserve. By integrating complementary spatial, temporal and kinematic dimensions within a unified evaluation strategy, the framework contributes to more comprehensive and interpretable assessment of pose estimation performance for neuroscience and biomedical movement analysis.

## Data Availability

All data, training scripts and evaluation pipelines are available upon request to the first author.
